# Motivation to COVID-19 Vaccination and Reasons for Hesitancy in Employees of a Czech Tertiary Care Hospital: A Cross-Sectional Survey

**DOI:** 10.3390/vaccines9080863

**Published:** 2021-08-05

**Authors:** Ladislav Štěpánek, Magdaléna Janošíková, Marie Nakládalová, Lubomír Štěpánek, Alena Boriková, Helena Vildová

**Affiliations:** 1 Department of Occupational Medicine, Faculty of Medicine and Dentistry, Palacký University Olomouc, I. P. Pavlova 185/6, 779 00 Olomouc, Czech Republic; magdalena.janosikova@fnol.cz (M.J.); marie.nakladalova@fnol.cz (M.N.); alena.borikova@fnol.cz (A.B.); helena.vildova@fnol.cz (H.V.); 2 Institute of Biophysics and Informatics, First Faculty of Medicine, Charles University, Salmovská 1, 120 00 Praha 2, Czech Republic; lubomir.stepanek@lf1.cuni.cz

**Keywords:** COVID-19, vaccination, motivation, hesitancy, vaccine safety, vaccine efficacy

## Abstract

High vaccination coverage among healthcare workers (HCWs) is crucial for managing the COVID-19 pandemic. The aim was to determine the demand for vaccination among all employees (*n* = 4553) of a tertiary care hospital after several weeks of the vaccine’s availability, and to analyze motives for acceptance and reasons for hesitancy through an anonymous online questionnaire. Upon the completion of data collection, the hospital’s vaccination coverage was at 69.8%. A total of 3550 completed questionnaires were obtained (2657 from vaccinated, 893 from unvaccinated employees). Significant predictors of vaccine acceptance were: age (odds ratio (OR) 1.01, 95% confidence interval (CI) 1.01–1.02), sex (OR (females) 0.58, 95% CI 0.45–0.75), job type (OR (non-physician HCWs) 0.54, 95% CI 0.41–0.72; OR (non-HCWs) 0.51, 95% CI 0.37–0.71), fear of COVID-19 (OR 1.4, 95% CI 1.34–1.46), history of COVID-19 (OR 0.41, 95% CI 0.34–0.49) and of influenza vaccination (OR 2.74, 95% CI 2.12–3.57). The most frequent motive for acceptance was the effort to protect family members (84%), while concerns about vaccine safety and side effects (49.4%), followed by distrust in the vaccine’s efficacy (41.1%) were the top reasons for hesitancy. To increase vaccination coverage among HCWs, it is necessary to raise awareness of vaccine safety and efficacy.

## 1. Introduction

A growing number of safe and effective vaccines represent an important tool, in combination with other measures, to protect people against the Coronavirus Disease 2019 (COVID-19), save lives and reduce widescale social disruption [1]. The course of vaccination against COVID-19 is currently constrained in many parts of the world by an insufficient supply of vaccines due to limited production capacities. However, even with sufficient efficacy and vaccine supplies, high vaccination coverage resulting from high participation rates is essential for the global society to be able to manage the pandemic [2,3].

Therefore, national COVID-19 vaccination strategies head towards the optimization of overall vaccine uptake and uptake by priority groups related to current vaccination phases [4]. Healthcare workers (HCWs) represent an irreplaceable resource in combating the COVID-19 pandemic, and, at the same time, suffer from much higher COVID-19 prevalence compared to the general population. HCWs have the ability to spread the disease easily. This makes them a top priority group for receiving COVID-19 vaccines globally [4,5].

Vaccination in the Czech Republic began in the last days of 2020—initially among HCWs, the oldest population group (aged 80+), and institutionalized persons. In mid-March, other groups of professions (the police, firefighters, teachers, etc.) were offered vaccination and the age threshold for the general population was lowered to 70+ years. Younger age groups and chronically ill persons were added gradually. Adults aged 50+ were granted access to vaccination at the beginning of May and adults aged 40+ in mid-May [6].

Knowing the underlying motivation for getting vaccinated is important for creating effective strategies. Mandatory vaccination is unlikely to be a viable option in individualistic societies. If vaccination hesitancy for COVID-19 is prevalent, then it is important to identify the motivational roots (i.e., reasons or attitudes) underlying the reluctance, and find ways to address these [7].

Accordingly, the aim of the present study was to map the demand for vaccination against COVID-19 among all employees of a tertiary care hospital with respect to their basic characteristics, several weeks after making vaccination available to all employees. The study also aimed to analyze the motivation for acceptance among the vaccinated and reasons for hesitancy among employees who are still vaccine reluctant.

## 2. Materials and Methods

### 2.1. Study Population

**Figure 1:**
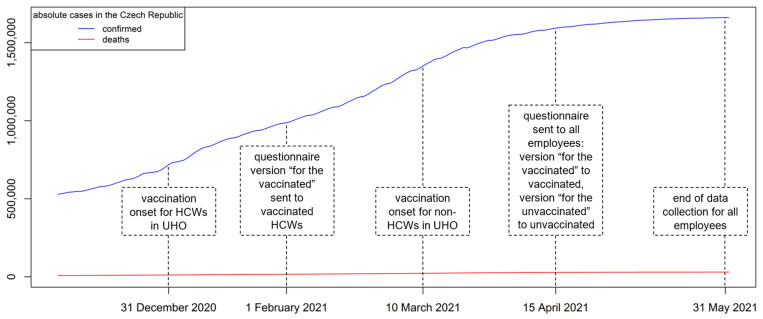
Timeline of data collection and the development of the pandemic in the Czech Republic. HCW, healthcare worker; UHO, University Hospital Olomouc.

The study population of this cross-sectional survey consisted of all employees of University Hospital Olomouc (UHO, *n* = 4553), who were asked to fill in an anonymous questionnaire distributed to individual hospital e-mail addresses. In UHO, HCWs had the opportunity to start vaccination from 31 December 2020, and non-HCWs from 10 March 2021. Data were collected from employees vaccinated with at least one dose of a vaccine against COVID-19 since the beginning of February 2021 (these employees received the questionnaire version “for the vaccinated”). Unvaccinated employees were invited to complete the questionnaire version “for the unvaccinated” at a time when the option to start vaccination had been open to all hospital employees, including non-HCWs, for 5 weeks and the number of new employees starting vaccination had already dropped significantly (to 10 per week). Employees were instructed not to complete the questionnaire more than once. Data collection for both groups ended on 31 May 2021. The timeline of data collection together with the development of the pandemic in the Czech Republic is summarized in Figure 1. A total of 97% of those vaccinated received the Comirnaty vaccine from Pfizer/BioNTech, the remaining 3% received vaccines from Moderna and AstraZeneca, whose first doses were applied only a few days in February.

### 2.2. Data Analysis

The collected data were statistically analyzed in the R software environment (R Foundation for Statistical Computing, Austria, Vienna; http://www.r-project.org/ (accessed on 21 July 2021)) using methods of descriptive statistics, including a chi-square test for pairwise comparisons. Comparisons of fear levels were performed using the analysis of variance (ANOVA). The level of statistical significance was set at *p* = 0.05. The Bonferroni correction was applied to adjust for multiple testing to minimize the risk of a type 1 error. Spearman’s correlation coefficient was determined between fear level and age. Binomial logistic regression was performed to examine the relationships between vaccine acceptance (being “vaccinated” or “unvaccinated” as a response variable) and other characteristics included in the responses of all employees (explanatory variables).

### 2.3. Survey Questionnaire

The Google form questionnaire used, which was based on a questionnaire applied in a study analyzing motivation for seasonal influenza vaccination by the same authors [8], contained mostly closed-ended questions on respondents’ personal information including sex, age and job type (i.e., physician, non-physician HCW, and non-HCW), health status (i.e., suffering from a chronic disease; a “yes” answer was followed by an open-ended question about the disease), already undergone COVID-19 (a “yes” answer was followed by a question about the date on which work incapacity ended), previous influenza vaccination at any time beforehand (a “yes” answer was followed by a question about the last year when vaccination took place), and a 10-point rating scale assessing the overall fear of COVID-19 itself. This section, unlike the following, was common for both vaccinated and unvaccinated employees.

Applying Maslow’s hierarchy of needs as the theoretical model, a multiple-choice pool of four possible motives for accepting and eight reasons for refusing the COVID-19 vaccine was developed (Table S1) using existing literature, expert evaluation and modification, and pilot testing of the questionnaire. The options were based on meeting physiological needs (e.g., self-protection), on meeting the need for safety, family protection, self-realization, and recognition [9].

## 3. Results

### 3.1. Basic Characteristics of Vaccinated and Unvaccinated Employees

**Figure 2:**
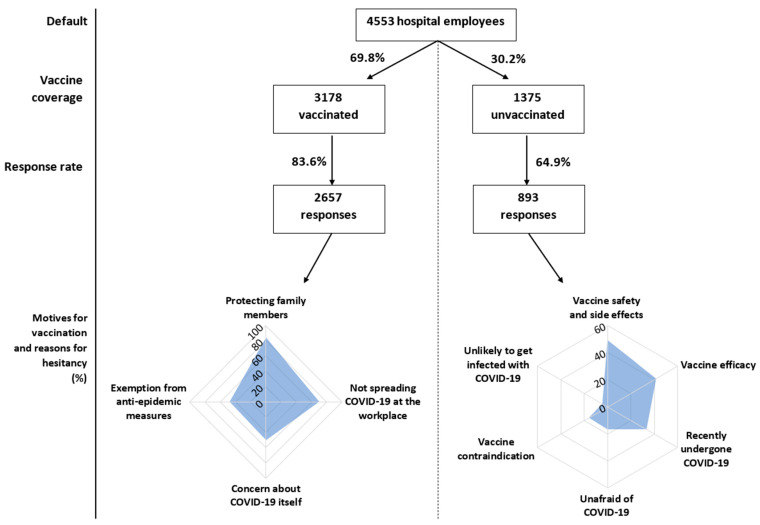
Study sample along with motives to vaccine acceptance and reasons for hesitancy.

A total of 4553 respondents yielded 3550 completed questionnaires, 2657 of which were from vaccinated and 893 from unvaccinated employees. The response rate was statistically significantly higher in vaccinated employees (83.6% vs. 64.9%; *p* < 0.001). The vaccination coverage of UHO employees at the time of completion of data collection was 69.8%. The characteristics of the respondents are given in Table 1 and Figure 2. The average age of vaccinated respondents was higher compared to the unvaccinated. Physicians represented almost a quarter of vaccinated respondents, which is about 14% more than among the unvaccinated. In contrast, there were 10% more non-physician HCWs among the unvaccinated. The share of non-HCWs also prevailed in the group of unvaccinated. The proportion of persons with a chronic disease did not differ significantly between the two groups. Almost a half of unvaccinated respondents, compared to only a quarter of vaccinated respondents, reported they had undergone the COVID-19 infection. Among all respondents, the highest proportion of a history of COVID-19 infection was in non-HCWs (36.4%), followed by non-physician HCWs (31.4%) and the lowest proportion was found among physicians (28.8%), but without the statistical significance of differences. Comparing termination dates of work incapacities showed that the gap between vaccinated and unvaccinated employees having had COVID-19 was 42 days (*p* < 0.001). In other words, unvaccinated respondents experienced COVID-19 more than a month later than the vaccinated. Table 1 also demonstrates significantly higher adherence to influenza vaccination, both at any time in the past and before the previous influenza season (2020/21), in the group of respondents already vaccinated against COVID-19.

The results gained from pairwise comparisons were confirmed by the regression analysis (Table 2). Growing age, fear of COVID-19 and history of influenza vaccination were factors positively associated with vaccination acceptance, while female sex, non-physician job type and history of COVID-19 were negatively associated.

Across the entire study sample, COVID-19 was perceived almost neutrally in terms of fear of this disease assessed using a 10-point scale (4.9 ± 2.4). Respondents vaccinated against COVID-19 showed a significantly higher level of fear than the unvaccinated (Table 1) and the association was also confirmed by the regression analysis. Among all respondents, regardless of acceptance or hesitancy towards COVID-19 vaccination, statistically significant differences in the assessment of fear were found in almost all subgroups, with the exception of job types and, after the Bonferroni correction, sex (Table 3). Thus, it is obvious that among the characteristics examined, the start of vaccination against COVID-19 and the anamnestic data on influenza vaccination were the factors determining the greatest scatter in the assessment of fear level. Correlations of fear level and age were very weak (r < 0.1) and statistically insignificant in the entire study sample and in both groups of respondents, vaccinated and unvaccinated.

### 3.2. Motivation to Vaccination Acceptance

The frequency of selecting various motives with respect to respondent subgroups is shown in Table 4 and Figure 2. The most frequently selected motive to the vaccination was the effort to protect family members, on the other hand, the least frequent motive was being exempted from anti-epidemic measures after vaccination. For this motive, and for the motive in the form of concern about COVID-19 itself, abundant statistically significant differences were recorded between respondent subgroups (Table 4). The elderly, chronically ill, or individuals vaccinated against influenza, as well as respondents without a history of COVID-19 infection, were more motivated by the concern about COVID-19 itself, but less often by the exemption from anti-epidemic measures after vaccination. Men compared to women and physicians compared to non-physicians selected both motives more frequently. The effort to prevent the spread of COVID-19 in the profession was, in terms of job types, a significantly stronger motivation for vaccination among physicians, weaker among non-physician HCWs, and weakest among non-HCWs. The effort to protect family members was chosen by 80–90% of respondents in all subgroups. Such a high percentage was achieved only in the case of the effort to prevent the spread of COVID-19 during profession performance among physicians. As far as other motives were concerned (open-ended question), the most repeated response was the effort to contribute to the solution to the entire pandemic (6.1%).

### 3.3. Reasons for Vaccination Hesitancy

Table 5 and Figure 2 introduce the frequency of selecting various reasons for hesitancy with respect to respondent subgroups. The most frequently selected reasons for not getting vaccinated were concerns about the vaccine’s safety and side effects, followed by distrust in the vaccine’s efficacy, and having previously undergone the COVID-19 infection. Younger respondents, non-physicians, and respondents without a history of COVID-19 were significantly more concerned about vaccine safety and side effects. These respondents, together with women and respondents never vaccinated against influenza, more often expressed distrust of the efficacy of vaccines. Men and individuals never vaccinated against influenza responded they were not afraid of COVID-19 more often. Chronically ill people were significantly more often discouraged from starting vaccination against COVID-19 due to vaccine contraindications. Respondents without a history of COVID-19 were less likely to be concerned about the infection, its course, and consequences, and more likely to be concerned about the efficacy, safety and side effects of the vaccine. The most frequent response under “other reasons for hesitancy” (open-ended question) was potential pregnancy (36 respondents).

## 4. Discussion

Vaccination coverage among all UHO employees reached approximately 70% at the time of completion of data collection. The minimum proportion of the vaccinated population required to achieve herd immunity against COVID-19 was estimated at 60–80%, particularly 72% for the Czech population [10]. Thus, despite the fact that the vaccine had been available for several weeks, only the minimum level was reached among employees of a tertiary care (university) hospital. HCWs are trusted sources of information who can influence an individual’s choice to accept the vaccine, therefore, their vaccine acceptance is of enormous importance [11]. The coverage in HCWs from various parts of the world certainly differs with regard to the availability of vaccines. Exact data on the vaccination coverage of HCWs from other countries are not yet available. 

Biswas et al. in their review of 35 available studies published until February 2021 revealed that the prevalence of COVID-19 vaccination hesitancy in HCWs worldwide ranged from 4.3 to 72% (average = 22.51% across all studies with 76,471 participants) [12]. A recent French multicenter study by Janssen et al. (*n* = 4349) noted that at least 22% of HCWs working in a hospital were not planning to get vaccinated at all [13], a study by Abuown et al. (*n* = 514) set this value at 24% in a London hospital [14]. Most of the studies found that individuals who were males, of older age, and doctoral degree holders (i.e., physicians) were more likely to accept COVID-19 vaccines. Factors such as direct care for patients and history of influenza vaccination were also found to increase COVID-19 vaccination uptake probability [12]. This corresponds to a statistically significantly higher proportion of men, older respondents, physicians, and persons previously vaccinated against influenza found among HCWs vaccinated against COVID-19 in our study, also confirmed by the logistic regression model. Similarly, and in accordance with our results, a British study by Hall et al. performed among HCWs working in a hospital (*n* = 23,324) explored that female, younger, non-physician and previously infected HCWs were less likely to get vaccinated [15]. Vaccination against COVID-19 was initiated in a situation of high daily numbers of reported cases in the Czech Republic (Figure 1). The epidemiological situation in UHO corresponded to the one in the Czech Republic with respect to the fact that the prevalence of COVID-19 among HCWs exceeded that in the general population [5]. In the last 4 months of 2020, 803 COVID-19 cases were recorded among UHO employees (i.e., 18% of all employees).

For a comprehensive understanding of the motives for vaccination, it is important to take into account the level of fear of COVID-19. Studies analyzing the fear of COVID-19 in HCWs in relation to vaccination uptake are not yet available in the literature, an exception being a Polish study by Szmyd et al., which revealed that willingness to get vaccinated was significantly strengthened by the growing fear of COVID-19 (odds ratio = 1.56, 95% confidence interval 1.43–1.7, *p* < 0.001). The authors of the questionnaire survey among 387 mostly hospital HCWs who had not started their vaccination also used a 10-point rating scale to assess fear, as was the case in the present study [16]. Our results confirmed a significantly higher level of fear of COVID-19 among respondents who had already started vaccination, compared to the unvaccinated group. Respondents without COVID-19 in their history showed a higher score for fear of the disease and were significantly more often vaccine-motivated by concerns about the disease itself than those who had already gone through the disease. According to the review by Biswas et al., a higher perceived risk of getting infected with COVID-19 was found to increase COVID-19 vaccination uptake probability [12]. Szmyd et al. found a statistically significantly higher assessment of fear among physicians compared to administrative healthcare assistants [16]. In contrast, significantly lower fear levels in physicians among other HCWs were detected in cross-sectional surveys by Prazeres et al. (*n* = 222) and Collantoni et al. (*n* = 996), using the Fear of COVID-19 scale [17,18]. There were no statistically significant differences in the fear levels depending on job types in our study.

As is the case for vaccination coverage, existing literature about motives for vaccination only offers data from studies performed before individuals begin their vaccination. With respect to analyzing motivation to get vaccinated against COVID-19, this means only intentions were analyzed, not data obtained early after the vaccine is available, as the present study is doing. From the point of view of Maslow’s hierarchy, the three most frequently chosen motives in our study represent family, patient, and self-protection. A study by Raftopoulos et al. amongst 2238 Greek and Cypriot HCWs observed that the main motives for those intending to get the COVID-19 vaccine included the protection of self (94.2%), family (98.7%), and patients (95.2%), as well as the mitigation of the COVID-19 pandemic (95.4%) [19]. Fares et al. in their study of 385 Egyptian HCWs identified reasons for vaccine acceptance to be risks of COVID-19 (93.8%), followed by the safety of the vaccine (57.5%), the effectiveness of the vaccine (56.3%), traveling facilitation (43.8%) [20]. In our work, as in the study by Raftopoulos et al., the most frequent motive for vaccination was family protection, followed by patient protection and self-protection. Concerns about a severe course of COVID-19 was a strong motive for vaccination against COVID-19 in a study by Hammer et al. carried out on the Finnish general population (*n* = 4151) [21].

Vaccine hesitancy (i.e., the delay in acceptance or refusal of the vaccine despite its availability) remains a pervasive issue in the general population as well as among HCWs across the globe [22]. Biswas et al. conclude in their review that the majority of studies found concerns about vaccine safety, efficacy, and potential side effects as top reasons for COVID-19 vaccination hesitancy [12]. Safety concerns were the dominant reason for vaccine rejection along with doubts about the availability of trial data and vaccine efficacy in the study by Abuown et al. [14]. Moreover, Szmyd et al. revealed as the main concern the development of long-term side effects after getting the COVID-19 vaccine [16]. Finally, the fear of side effects of the vaccine was also the reason for vaccine hesitancy in the study by Fares et al. [20]. Concerns about vaccine safety, side effects and vaccine efficacy were the most frequent reasons for vaccine hesitancy in our study, too, reported by almost half of unvaccinated respondents. In the group of unvaccinated employees, one-third of respondents cited as a reason for being hesitant that they had already undergone COVID-19 and assumed a lasting immunity. The frequent choice of this reason for not initiating vaccination yet corresponds to a statistically significantly higher relative incidence of COVID-19 in the group of unvaccinated employees as well as to on average a later date of termination of work incapacity for COVID-19. Many unvaccinated employees who have previously suffered COVID-19 can be expected to get vaccinated in the coming months, and adjustments to the vaccination guidelines regarding pregnancy may lead to higher willingness to get vaccinated in some women too. However, at the time of the manuscript submission, vaccination coverage at the hospital increased by only about 1.4% compared to the end of data collection (mostly among physicians).

To improve the acceptance of COVID-19 vaccination, it is imperative to implement multi-level strategies (population-wide, organization-wide, inter-individual, and individual), along with evidence-based strategies that address vaccine hesitancy. Equally critical is the widespread adoption of evidence-based best practices that were developed and refined with previous vaccines [23]. Jarrett et al., in their systematic review, focused on previous vaccine strategies addressing hesitancy with the evaluation of their impact on vaccination uptake. The review revealed that dialogue-based or multi-component interventions were most effective. The dialogue-based intervention included involving leaders (religious or traditional), social mobilization, social media, mass media, and communication or information-based tools for HCWs. However, given the complexity of vaccine hesitancy and the novelty of the COVID-19 pandemic, strategies should be carefully tailored to the target population and their respective reasons for hesitancy. Strategies must also be self-reflective and adaptive, assessing progress and outcomes and reevaluating strategies as needed. The COVID-19 pandemic was accompanied by an overabundance of misinformation, which should be reflected in developing trusted sources, responding to misinformation, and building people’s resilience to misinformation [24].

To the best of our knowledge, studies analyzing motivation for getting vaccinated against COVID-19 retrospectively and exploring the reasons for vaccine hesitancy among HCWs are scarce in the literature, which limits our options to compare results. Another limitation of the study is a failure to take into account the time lag between undergone COVID-19 and completing the questionnaire. Response rates between vaccinated and unvaccinated employees differed with high statistical significance. However, fixed effects, whether percentage estimates or logistic regression, are robust against these differences. We performed the best-case and worst-case scenario simulations without substantial differences in the reported results. However, higher response rates would probably bring partial refinements. In the present study, a simple 10-point rating scale was used to assess fear, as the inclusion of some validated scales (e.g., the Fear of COVID-19 scale) could lead to a lower willingness to participate in the survey due to their time-consuming nature. A high number of respondents partially compensates for the absence of validated questionnaires focused on fear analysis. The changing course of the pandemic, information on new mutations and new findings on vaccines may have influenced vaccination coverage and questionnaire responses, however, this should be reduced by the use of vaccines almost exclusively from Pfizer/BioNTech, whose efficacy and safety were not questioned as often as other vaccines.

## 5. Conclusions

The vaccination coverage of all employees of a large hospital even after weeks of a lasting possibility to start vaccination against COVID-19 reached only 70%. Physicians demanded the vaccination more than non-physicians. The most frequent motive for vaccination was family protection (84%), followed by patient protection (69.7%), self-protection (50.2%) and exemption from anti-epidemic measures (48%). The most frequent reasons for hesitancy were concerns about vaccine safety and side effects (49.4%), distrust of vaccine efficacy (41.1%) and history of COVID-19 infection (33.4%). For increasing vaccination coverage, information campaigns should address these main motives for acceptance and hesitancy. This means improving awareness of the safety of COVID-19 vaccines and, at the same time, of their efficacy and the ability to protect, especially family members.

## Figures and Tables

**Table 1:** Characteristics of vaccinated and unvaccinated respondents.

	Whole Sample	Vaccinated	Unvaccinated	*p*-Value
Addressed employees (No.)	4553	3178 (69.8%)	1375 (30.2%)	-
**Respondents** (No., %)	**3550** (78%)	**2657** (83.6%)	**893** (64.9%)	<0.001
Age (years; average ± SD)	43.2 ± 11.3	43.6 ± 11.2	41.9 ± 11.5	<0.001
Level of fear of COVID-19 (average ± SD)	4.9 ± 2.4	5.3 ± 2.3	3.5 ± 2.3	<0.001
**Respondent subgroups**				
Females (No., %)	2791 (78.6%)	2020 (76%)	771 (86.3%)	<0.001
Males (No., %)	759 (21.4%)	637 (24%)	122 (13.7%)	<0.001
Physicians (No., %)	764 (21.5%)	662 (24.9%)	102 (11.4%)	<0.001
Non-physician HCWs (No., %)	2234 (62.9%)	1601 (60.3%)	633 (70.9%)	<0.001
Non-HCWs (No., %)	552 (15.5%)	394 (14.8%)	158 (17.7%)	0.041
With a chronic disease (No., %)	1069 (30.1%)	807 (30.4%)	262 (29.3%)	0.56
History of COVID-19 (No., %)	1122 (31.6%)	709 (26.7%)	413 (46.2%)	<0.001
Influenza vaccination at any time in the past (No., %)	909 (25.6%)	824 (31%)	85 (9.5%)	<0.001
Influenza vaccination before the season 2020/21 (No., %)	670 (18.9%)	620 (23.3%)	50 (5.6%)	<0.001

SD, standard deviation; HCW, healthcare worker.

**Table 2:** Predictors of vaccine acceptance.

Characteristics	Odds Ratio	95% Confidence Interval	Standard Error	*p*-Value
Sex (females)	0.58	0.45; 0.75	−4.1	<0.001
Age	1.01	1.01; 1.02	3.57	<0.001
Level of fear of COVID-19	1.4	1.34; 1.46	16.08	<0.001
Non-physician HCWs vs. physicians	0.54	0.41; 0.72	−4.26	<0.001
Non-HCWs vs. physicians	0.51	0.37; 0.71	−3.98	<0.001
Chronic disease	0.8	0.66; 0.97	−2.26	0.054
History of COVID-19	0.41	0.34; 0.49	−9.96	<0.001
Influenza vaccination at any time in the past	2.74	2.12; 3.57	7.58	<0.001

HCW, healthcare worker.

**Table 3:** Level of fear of COVID-19 in the entire respondent sample regardless of vaccination against COVID-19.

Subgroups	Fear Level (±SD)	*p*-Value
Females	4.9 ± 2.5	0.022
Males	4.7 ± 2.3	
Physicians	5 ± 2.4	0.356
Non-physician HCWs	4.9 ± 2.4	
Non-HCWs	5 ± 2.5	
With a chronic disease	5.3 ± 2.5	<0.001 ^†^
Without a chronic disease	4.7 ± 2.4	
History of COVID-19	4.7 ± 2.3	0.001 ^†^
No history of COVID-19	5 ± 2.5	
Influenza vaccination at any time in the past	5.6 ± 2.3	<0.001 ^†^
Never vaccinated against influenza	4.6 ± 2.4	

SD, standard deviation; HCW, healthcare worker. ^†^ statistically significant after the Bonferroni correction.

**Table 4:** Motives to get vaccinated against COVID-19 (multiple-choice options) and the frequency of their selection.

	Concerns about COVID-19 Itself	An Effort to Prevent the Spread of COVID-19 during the Performance of My Profession	An Effort to Protect Family Members	Being Exempted from Restrictive Anti-Epidemic Measures after Vaccination
All respondents	1334 (50.2%)	1853 (69.7%)	2232 (84%)	1276 (48%)
**Respondent subgroups**				
Younger than the age median (<44 years)	568 (43.1%) *^,†^	928 (70.4%)	1134 (86%) *	732 (55.5%) *^,†^
Older than the age median (≥44 let)	766 (57.2%)	925 (69.1%)	1098 (82.1%)	544 (40.7%)
Females	986 (48.8%) *	1381 (68.4%) *	1692 (83.8%)	922 (45.6%) *^,†^
Males	348 (54.6%)	472 (74.1%)	540 (84.8%)	354 (55.6%)
Physicians	372 (56.2%) *	535 (80.8%) *^,†^	566 (85.5%)	384 (58%) *^,†^
Non-physician HCWs	760 (47.5%)	1101 (68.8%)	1349 (84.3%)	730 (45.6%)
Non-HCWs	202 (51.3%)	217 (55.1%)	317 (80.5%)	162 (41.1%)
With a chronic disease	476 (59%) *^,†^	567 (70.3%)	689 (85.4%)	330 (40.9%) *^,†^
Without a chronic disease	858 (46,4%)	1286 (69.5%)	1543 (83.4%)	946 (51.1%)
History of COVID-19	310 (43.7%) *^,†^	504 (71.1%)	603 (85%)	372 (52.5%)
No history of COVID-19	1024 (52.6%)	1349 (69.3%)	1629 (83.6%)	904 (46.4%)
Influenza vaccination at any time in the past	492 (59.7%) *^,†^	615 (74.6%) *^,†^	717 (87%) *	373 (45.3%)
Never vaccinated against influenza	842 (45.9%)	1238 (67.5%)	1515 (82.7%)	903 (49.3%)

HCW, healthcare worker. * *p* < 0.05, ^†^ statistically significant after the Bonferroni correction.

**Table 5:** Reasons for hesitancy to get vaccinated against COVID-19 (multiple-choice options) and the frequency of their selection.

	I Am Not Afraid of COVID-19-Its Course and Consequences	I Do Not Find Getting Infected with COVID-19 Likely	I Do Not Trust the Efficacy of Vaccines against COVID-19	I Have Concerns about the Safety and Side Effects of Vaccines against COVID-19	I Went through COVID-19 (and Assume Lasting Immunity against the Disease)	I Have Contraindications or Expect a Complicated Vaccination Course in My Case
All respondents	167 (18.7%)	40 (4.5%)	370 (41.1%)	441 (49.4%)	298 (33.4%)	144 (16.1%)
**Respondent subgroups**						
Younger than the age median (≤43 le)	87 (19%)	24 (5.2%)	209 (45.5%) *	251 (54.7%) *^,†^	136 (29.6%) *	66 (14.4%)
Older than the age median (>43 let)	80 (18.4%)	16 (3.7%)	161 (37.1%)	190 (43.8%)	162 (37.3%)	78 (18%)
Females	133 (17.3%) *	32 (4.2%)	333 (43.2%) *	390 (50.6%)	253 (32.8%)	126 (16.3%)
Males	34 (27.9%)	8 (6.6%)	37 (30.3%)	51 (41.8%)	45 (36.9%)	18 (14.8%)
Physicians	14 (13.7%)	8 (7.8%)	14 (13.7%) *^,†^	30 (29.4%) *^,†^	32 (31.4%)	12 (11.8%)
Non-physician HCWs	122 (19.3%)	26 (4.1%)	293 (46.3%)	347 (54.8%)	203 (32.1%)	108 (17.1%)
Non-HCWs	31 (19.6%)	6 (3.8%)	63 (39.9%)	64 (40.5%)	63 (39.9%)	24 (15.2%)
With a chronic disease	46 (17.6%)	4 (1.5%) *	99 (37.8%)	122 (46.6%)	81 (30.9%)	78 (29.8%) *^,†^
Without a chronic disease	121 (19.2%)	36 (5.7%)	271 (42.9%)	319 (50.6%)	217 (34.4%)	66 (10.5%)
History of COVID-19	56 (13.6%) *^,†^	4 (1%) *^,†^	134 (32.4%) *^,†^	165 (40%) *^,†^	287 (69.5%) *^,†^	64 (15.5%)
No history of COVID-19	111 (23.1%)	36 (7.5%)	236 (49.2%)	276 (57.5%)	11 (2.3%)	80 (16.7%)
Influenza vaccination at any time in the past	5 (5.9%) *^,†^	2 (2.4%)	26 (30.6%) *	36 (42.4%)	33 (38.8%)	10 (11.8%)
Never vaccinated against influenza	162 (20%)	38 (4.7%)	344 (42.6%)	405 (50.1%)	265 (32.8%)	134 (16.6%)

HCW, healthcare worker. * *p* < 0.05, ^†^ statistically significant after the Bonferroni correction.

## Data Availability

The data presented in this study are available from the corresponding author upon reasonable request.

## Supplementary Materials

The following are available online at https://www.mdpi.com/article/10.3390/vaccines9080863/s1, Table S1: The questionnaire.

## References

[1] World Health Organization COVID-19 Vaccines. https://www.who.int/westernpacific/emergencies/covid-19/covid-19-vaccines.

[2] Hughes K., Gogineni V., Lewis C., Deshpande A. (2021). Considerations for fair prioritization of COVID-19 vaccine and its mandate among healthcare personnel. Curr. Med. Res. Opin..

[3] Wouters O.J., Shadlen K.C., Salcher-Konrad M., Pollard A.J., Larson H.J., Teerawattananon Y., Jit M. (2021). Challenges in ensuring global access to COVID-19 vaccines: Production, affordability, allocation, and deployment. Lancet.

[4] European Centre for Disease Prevention and Control Overview of the Implementation of COVID-19 Vaccination Strategies and Deployment Plans in the EU/EEA. https://www.ecdc.europa.eu/en/publications-data/overview-implementation-covid-19-vaccination-strategies-and-deployment-plans.

[5] Gholami M., Fawad I., Shadan S., Rowaiee R., Ghanem H., Hassan Khamis A., Ho S.B. (2021). COVID-19 and healthcare workers: A systematic review and meta-analysis. Int. J. Infect. Dis..

[6] Ministry of the Interior of the Czech Republic Vaccination Timeline. https://covid.gov.cz/en/situations/register-vaccination/vaccination-timeline.

[7] Taylor S., Landry C.A., Paluszek M.M., Groenewoud R., Rachor G.S., Asmundson G.J.G. (2020). A Proactive Approach for Managing COVID-19: The Importance of Understanding the Motivational Roots of Vaccination Hesitancy for SARS-CoV2. Front. Psychol..

[8] Štěpánek L., Nakládalová M., Vildová H., Boriková A., Janošíková M., Ivanová K. (2021). Demand and motivation for influenza vaccination among healthcare workers before and during the COVID-19 era: A cross-sectional survey. Hum. Vaccines Immunother..

[9] Taormina R.J., Gao J.H. (2013). Maslow and the motivation hierarchy: Measuring satisfaction of the needs. Am. J. Psychol..

[10] Kwok K.O., Lai F., Wei W.I., Wong S.Y.S., Tang J.W.T. (2020). Herd immunity—Estimating the level required to halt the COVID-19 epidemics in affected countries. J. Infect..

[11] Gabarda A., Butterworth S.W. (2021). Using Best Practices to Address COVID-19 Vaccine Hesitancy: The Case for the Motivational Interviewing Approach. Health Promot. Pract..

[12] Biswas N., Mustapha T., Khubchandani J., Price J.H. (2021). The Nature and Extent of COVID-19 Vaccination Hesitancy in Healthcare Workers. J. Community Health.

[13] Janssen C., Maillard A., Bodelet C., Claudel A.L., Gaillat J., Delory T., On Behalf of the Acv Alpin Study Group (2021). Hesitancy towards COVID-19 Vaccination among Healthcare Workers: A Multi-Centric Survey in France. Vaccines.

[14] Abuown A., Ellis T., Miller J., Davidson R., Kachwala Q., Medeiros M., Mejia K., Manoraj S., Sidhu M., Whittington A.M. (2021). COVID-19 vaccination intent among London healthcare workers. Occup. Med..

[15] Hall V.J., Foulkes S., Saei A., Andrews N., Oguti B., Charlett A., Wellington E., Stowe J., Gillson N., Atti A. (2021). COVID-19 vaccine coverage in health-care workers in England and effectiveness of BNT162b2 mRNA vaccine against infection (SIREN): A prospective, multicentre, cohort study. Lancet.

[16] Szmyd B., Karuga F.F., Bartoszek A., Staniecka K., Siwecka N., Bartoszek A., Błaszczyk M., Radek M. (2021). Attitude and Behaviors towards SARS-CoV-2 Vaccination among Healthcare Workers: A Cross-Sectional Study from Poland. Vaccines.

[17] Prazeres F., Passos L., Simões J.A., Simões P., Martins C., Teixeira A. (2020). COVID-19-Related Fear and Anxiety: Spiritual-Religious Coping in Healthcare Workers in Portugal. Int. J. Environ. Res. Public Health.

[18] Collantoni E., Saieva A.M., Meregalli V., Girotto C., Carretta G., Boemo D.G., Bordignon G., Capizzi A., Contessa C., Nesoti M.V. (2021). Psychological Distress, Fear of COVID-19, and Resilient Coping Abilities among Healthcare Workers in a Tertiary First-Line Hospital during the Coronavirus Pandemic. J. Clin. Med..

[19] Raftopoulos V., Iordanou S., Katsapi A., Dedoukou X., Maltezou H.C. (2021). A comparative online survey on the intention to get COVID-19 vaccine between Greek and Cypriot healthcare personnel: Is the country a predictor?. Hum. Vaccines Immunother..

[20] Fares S., Elmnyer M.M., Mohamed S.S., Elsayed R. (2021). COVID-19 Vaccination Perception and Attitude among Healthcare Workers in Egypt. J. Prim. Care Community Health.

[21] Hammer C.C., Cristea V., Dub T., Sivelä J. (2021). High but slightly declining COVID-19 vaccine acceptance and reasons for vaccine acceptance, Finland April to December 2020. Epidemiol. Infect..

[22] Dzieciolowska S., Hamel D., Gadio S., Dionne M., Gagnon D., Robitaille L., Cook E., Caron I., Talib A., Parkes L. (2021). Covid-19 vaccine acceptance, hesitancy, and refusal among Canadian healthcare workers: A multicenter survey. Am. J. Infect. Control.

[23] Finney Rutten L.J., Zhu X., Leppin A.L., Ridgeway J.L., Swift M.D., Griffin J.M., St Sauver J.L., Virk A., Jacobson R.M. (2021). Evidence-Based Strategies for Clinical Organizations to Address COVID-19 Vaccine Hesitancy. Mayo Clin. Proc..

[24] Jarrett C., Wilson R., O’Leary M., Eckersberger E., Larson H.J. (2015). SAGE Working Group on Vaccine Hesitancy. Strategies for addressing vaccine hesitancy—A systematic review. Vaccine.

