# Supplementary material for: Motivation to COVID-19 Vaccination and Reasons for Hesitancy in Employees of a Czech Tertiary Care Hospital: A Cross-Sectional Survey
Source: Vaccines (Basel). 2021 Aug 5;9(8):863. doi: 10.3390/vaccines9080863 (PMC8402579; doi:10.3390/vaccines9080863)
Supplement: Supplementary file 1 [file vaccines-09-00863-s001.zip › vaccines-1299584-supplementary.pdf]

Motivation to the vaccination against COVID-19/reasons for not getting vaccinated yet among employees of University Hospital Olomouc – the questionnaire

Common part for both the vaccinated and unvaccinated:

Sex:

- male
- female

Age (years):

What is your job type in University Hospital Olomouc?

- Physician
- Non-physician (but healthcare worker)
- Non-healthcare worker

Do you suffer from a chronic disease?

- yes
- no

If you suffer from a chronic disease, what is it?

- cardiovascular disease
- arterial hypertension
- kidney disease
- liver disease
- respiratory tract disease
- immune system disease
- diabetes mellitus
- endocrine system disease
- anemia
- other:

Have you undergone COVID-19?

- yes
- no

If you have undergone COVID-19, your work incapacity ended on (date):

Have you ever been vaccinated against seasonal influenza?

- yes
- no

If you have been vaccinated against seasonal influenza, your last vaccination was (year):

Try to assess your overall fear of COVID-19 (its course, consequences...) on a scale of 1-10:

1 – I am not afraid of COVID-19; 10 – I have serious concerns about COVID-19

Version for the vaccinated:

I was motivated to get vaccinated against COVID-19 by (choose any number of options):

- Concerns about COVID-19 itself

- An effort to prevent the spread of COVID-19 during the performance of my profession
- An effort to protect family members
- Being exempted from restrictive anti-epidemic measures after vaccination
- Other:

Version for the unvaccinated:

Reasons why I haven't been vaccinated against COVID-19 yet (choose any number of options):

- I am not afraid of COVID-19 – its course and consequences
- I do not find getting infected with COVID-19 likely
- I do not trust the efficacy of vaccines against COVID-19
- I have concerns about the safety and side effects of vaccines against COVID-19
- I went through COVID-19 (and assume lasting immunity against the disease)
- I have contraindications or expect a complicated vaccination course in my case
- Other:
